# LEARN 2 MOVE 0-2 years: effects of a new intervention program in infants at very high risk for cerebral palsy; a randomized controlled trial

**DOI:** 10.1186/1471-2431-10-76

**Published:** 2010-11-02

**Authors:** Tjitske Hielkema, Elisa G Hamer, Heleen A Reinders-Messelink, Carel GB Maathuis, Arend F Bos, Tineke Dirks, Lily van Doormaal, Johannes Verheijden, Carla Vlaskamp, Eline Lindeman, Mijna Hadders-Algra

**Affiliations:** 1 Beatrix Children's Hospital - Division of Developmental Neurology, University Medical Center Groningen, Groningen, The Netherlands; 2 Department of Rehabilitation Medicine, University Medical Center Groningen, Groningen, The Netherlands; 3 Rehabilitation Center 'Revalidatie Friesland', Beetsterzwaag, The Netherlands; 4 Beatrix Children's Hospital - Division of Neonatology, University Medical Center Groningen, Groningen, The Netherlands; 5 BOSK, Association of physically disabled persons and their parents, Utrecht, The Netherlands; 6 Department of Behavioural and Social Sciences, Rijksuniversiteit Groningen, Groningen, The Netherlands; 7 Rudolf Magnus Institute of Neuroscience and Centre of Excellence for Rehabilitation medicine, University Medical Center Utrecht and Rehabilitation Center De Hoogstraat, Utrecht, The Netherlands

## Abstract

**Background:**

It is widely accepted that infants at risk for cerebral palsy need paediatric physiotherapy. However, there is little evidence for the efficacy of physiotherapeutic intervention. Recently, a new intervention program, COPCA (Coping with and Caring for infants with special needs - a family centered program), was developed. COPCA has educational and motor goals. A previous study indicated that the COPCA-approach is associated with better developmental outcomes for infants at high risk for developmental disorders. LEARN 2 MOVE 0-2 years evaluates the efficacy and the working mechanisms of the COPCA program in infants at very high risk for cerebral palsy in comparison to the efficacy of traditional infant physiotherapy in a randomized controlled trial. The objective is to evaluate the effects of both intervention programs on motor, cognitive and daily functioning of the child and the family and to get insight in the working elements of early intervention methods.

**Methods/design:**

Infants are included at the corrected age of 1 to 9 months and randomized into a group receiving COPCA and a group receiving traditional infant physiotherapy. Both interventions are given once a week during one year. Measurements are performed at baseline, during and after the intervention period and at the corrected age of 21 months. Primary outcome of the study is the Infant Motor Profile, a qualitative evaluation instrument of motor behaviour in infancy. Secondary measurements focus on activities and participation, body functions and structures, family functioning, quality of life and working mechanisms. To cope with the heterogeneity in physiotherapy, physiotherapeutic sessions are video-recorded three times (baseline, after 6 months and at the end of the intervention period). Physiotherapeutic actions will be quantified and related to outcome.

**Discussion:**

LEARN 2 MOVE 0-2 years evaluates and explores the effects of COPCA and TIP. Whatever the outcome of the project, it will improve our understanding of early intervention in children with cerebral palsy. Such knowledge is a prerequisite for tailor-made guidance of children with CP and their families.

**Trial registration:**

The trial is registered under NTR1428.

## Background

Cerebral Palsy (CP) is the most common cause of physical disability in pediatric rehabilitation [1]. Little evidence exists that current interventions are effective in optimizing daily life functioning [2]. LEARN 2 MOVE 0-2 (L2M 0-2) is part of the Dutch national LEARN 2 MOVE research program [3-5], which evaluates new interventions in rehabilitation for children and adolescents with CP in different age cohorts.

Because the diagnosis CP requires an age of at least 18 months [6,7], L2M 0-2 focuses on infants at high risk for CP. Neurological findings, the presence of a lesion of the brain or other factors may indicate that infants are at risk for CP. It is generally assumed that infants at risk for CP need physiotherapy. Theoretically, intervention at early age when the brain is very plastic, should be more effective than intervention which starts beyond infancy [8]. However, knowledge on the efficacy of early intervention is limited. Systematic reviews indicated that early intervention programs may have a positive effect on cognitive development of young children, but no or minimal effect on motor development [2,9,10]. Interestingly, it is unknown which elements of intervention lead to improvement in function. The limited evidence for efficacy of current interventions led to the development of a new intervention program, COPCA (Coping with and Caring for Infants with special needs - a family centered program [11], unpublished data Dirks et al.). COPCA is based on new insights in the field of education and family care [12,13] and the motor developmental principles of the Neuronal Group Selection Theory (NGST) [14,15]. The COPCA intervention has been evaluated in the Groningen VIP (Vroege Interventie Project) study. At RCT-level, COPCA was associated with a minimally better cognitive development than traditional infant physiotherapy (TIP), but the two interventions groups did not differ in traditional measures of motor development until 18 months. After process analyses of physiotherapeutic sessions, COPCA-based physiotherapeutic actions were associated with improved functional ability and better motor outcome. This was true in particular for infants who developed CP [16] (unpublished data Blauw-Hospers et al.). These first findings on a possibly beneficial effect of the COPCA-program encouraged us to further investigate the effects of COPCA in a population at very high risk for CP. The framework of LEARN 2 MOVE 0-2 offered this possibility.

## Methods/design

The objective of L2M 0-2 is to evaluate the efficacy and working mechanisms of the new intervention program COPCA in infants at very high risk for developing CP. To evaluate the efficacy, the COPCA approach will be compared with regular care, traditional infant physiotherapy (TIP), in a randomized controlled trial (RCT).

We will recruit 40 infants at very high risk for cerebral palsy. The study is coordinated by the research team of the University Medical Center Groningen (UMCG). The Medical Ethics Committee of the UMCG granted approval for the study. The trial is registered under NTR1428. Participation is voluntary and participants can withdraw at any time without affecting regular treatment. The intervention period is 12 months in duration and has no known risks for participants.

### Study sample

Forty infants will be recruited via their treating physician or physiotherapist. The infants will be recruited in the provinces of Groningen, Friesland, Drenthe, Overijssel and in and around the city of Amsterdam. In these regions 24 hospitals are located.

### Inclusion criteria

Infants aged 1 to 9 months corrected age (CA) at high risk for developing CP, based on the presence of one of the following:

a) Cystic periventricular leukomalacia, diagnosed on serial ultrasound assessments of the brain [17].

b) Unilateral or bilateral parenchymal lesion of the brain [18].

c) Term/near-term asphyxia resulting in Sarnat 2 or 3 [19] with brain lesions on MRI and/or with neurological dysfunction during infancy suggesting the development of CP.

d) Neurological dysfunction suggestive of development of CP.

### Exclusion criteria

Infants are excluded on the basis of one of the following criteria:

a) An additional severe congenital disorder, such as serious congenital heart disorder.

b) Caregivers have an insufficient understanding of the Dutch language.

### Power calculation

Power calculation based on the primary outcome measure, the Infant Motor Profile (IMP [20]; see measurements) indicates that two groups of 19 infants result in a power of 80% (α = 0.05) to detect a clinically relevant change of 7.5 points in the total IMP score (SD = 8.2).

### Recruitment procedure

Paediatricians, physiatrists and physiotherapists are informed about the study by both written and oral information. Information is published in various local and national journals directed at professionals and caregivers.

The treating physician or physiotherapist informs eligible families about the study and informs the research-team in the UMCG about them. If caregivers are interested in the study, an information letter is sent and they are free to ask more information. If caregivers decide to participate, an assignment form is sent back. Randomisation will take place in blocks stratified according to the type of brain lesion or neurological dysfunction. Infants are assigned to one of the two interventions and baseline measurements will start. Parents, caregivers and therapists can not be blinded with respect to type of intervention. Assessors will be blinded with respect to group allocation.

### Intervention

Infants participating in the study will receive either COPCA or TIP. Intervention is carried out in the infant's home and coordinated by the UMCG-team; evaluation is carried out by the UMCG-team. COPCA will be provided by therapists with a specific training in COPCA, TIP by paediatric physiotherapists selected by the paediatrician in charge of the child's care. Intervention will be provided once a week during a year.

### COPCA

COPCA is a family relationship oriented program. COPCA aims to promote activities and participation of the infant with special needs and its family, taking into account the limitations imposed by bodily impairments. COPCA consists of two components:

a) A family involvement and educational component, based on recent insights in the family and educational field [12,13]. Important aspects are family autonomy and rearing the child from the family's own educational perspective [21,22].

b) A neurodevelopmental component based on the principles of the Neuronal Group Selection Theory [14,15]. Important aspects are variation and variability, aiming to increase the infant's motor repertoire and improved ability to select a specific strategy fit for function in a specific daily life situation.

In COPCA principles of coaching are used to promote creative exploration of the competencies of the family members including the infant with special needs in order to stimulate self-made decisions and improve the quality of life. The physiotherapist, called coach, listens, informs and observes while the caregiver is involved in daily routines with the child, including play, thereby creating a situation in which caregivers feel free to explore and discuss alternative strategies. Key words of COPCA intervention are variation, exploration, trial and error, self produced motor behaviour (no 'hands on'), coaching (no training), family autonomy and family rituals (unpublished data Dirks et al).

### TIP

Control therapy is TIP, which - in the Netherlands - is based to a large extent on the 'living concept' of NeuroDevelopmental Treatment (NDT) [23]. TIP based on NDT focuses primarily on limitations imposed by bodily impairments and functional activities of the infant with special needs. The two major components are:

a) Neurodevelopmental principles, consisting of a mix of neuromaturational assumptions, sensorimotor problem solving strategies and the principles of the dynamic systems theory. The therapist plays a key role in teaching and instructing these principles. By providing sensorimotor experiences, the therapist learns the infant to engage in developmental activities.

b) Family members are seen as 'the most important people on the baby's team' in the planning of treatment goals, according to Bly [24].

Typical development is the framework for treatment in NDT. Problem solving is used to identify missing or atypical elements of functional movements and posture. The therapist treats the infant and selects during the treatment handling (hands on) strategies to facilitate and prepare the infant for age specific function. The caregivers are instructed how to continue and integrate these treatment strategies, which often involve hands-on techniques, into daily life. For the implementation of NDT in daily practice this means that a large repertoire of facilitation techniques like handling are used to reduce atypical functional activities and to prepare the infant for optimally independent function (unpublished data Dirks et al.). Due to the different influences which are incorporated in TIP a large heterogeneity in treatment application exists [25].

### Measurements (table 1)

**Table 1:** Measurements LEARN 2 MOVE 0-2

Instrument	Baseline (T0)	After 3 mo (T1)	After 6 mo (T2)	After 12 mo (T3)	At 21 mo CA*
Primary Outcome					

◦ IMP	+	+	+	+	+

Secondary Outcome, child					

◦ Neurological assessment	+	+	+	+	+

◦ AIMS	+	+	+	+	+

◦ GMFM	+	+	+	+	+

◦ Bayley PDI	+	+	+	+	+

◦ Bayley MDI	+	+	+	+	+

◦ VABS (P)	+		+	+	

◦ PEDI (P)	+			+	

◦ ITQOL (P)	+			+	

Secondary Outcome, family					

◦ Video parent child interaction	+		+	+	

◦ Utrechtse Coping List (P)	+			+	

◦ NOSI-K (P)	+			+	

◦ RDI	+	+	+	+	+

◦ MPOC (P), MPOC-SP (T)	+			+	

◦ FES (P)	+		+	+	

◦ CBS list QoL (P)	+		+	+	

Working mechanisms					

◦ Assessment postural control	+		+	+	+

◦ Video therapeutic session	+ #		+	+	

◦ DAIS	+		+	+	

◦ Weekly diaries, parents	+	+	+	+	

◦ Weekly diaries, therapists	+	+	+	+	+

CA = corrected age, the additional assessment at 21 months corrected age is scheduled for infants who enter the study before the corrected age of 8 months. The assessment is required to determine in all infants the diagnosis of CP as good as possible.

# The first video of a therapeutic session is made 1 month after the onset of intervention.

P = parental questionnaire or interview, this means that for the three child outcome measures it are assessments by means of proxy; T = assessment of therapist, i.e. service provider

Primary outcome focuses on the performance of mobility-related activities measured by the Infant Motor Profile (IMP) [20,26], secondary measures on participation and quality of life of participants and parents and on body functions and structures. Secondary outcomes assess child-related as well as caregiver-related variables. Possible effect modifiers such as medical history, demographic variables and compliance of therapists and caregivers will be taken into account.

The infants will be assessed at inclusion (T0), and at 3, 6 and 12 months after the onset of the intervention (T1, T2, T3). In infants included prior to the age of 8 months, an additional assessment is scheduled at the corrected age of 21 months (T4). Assessments will take place at infants' home and/or at the UMCG.

Primary outcome is motor performance as measured by the IMP, a video-based assessment which provides information on a child's motor repertoire and its ability to adapt motor behaviour to the specifics of the situation [20].

#### Secondary child related outcomes

1. Neurological condition, according to the Touwen Infant Neurological Examination [27,28], in order to specify the neurological condition of the child, for instance the absence or presence of CP.

2. Measurements of infant motor skills:

a. AIMS (Alberta Infant Motor Scales). The AIMS is an instrument designed to assess gross motor development during infancy in infants with typical and atypical development. It has a good reliability and validity [29], but in older infants the AIMS suffers from ceiling effects.

b. GMFM (Gross Motor Function Measure) [30]. The GMFM is designed to assess gross motor development in children with CP. It has good reliability and validity but in infants it suffers from bottom effects. The latter also implies that at early age or in children with severe motor dysfunction only a few items can be performed.

c. Bayley Scales of Infant Development (BSID-II), Psychomotor Development Index (PDI) which measures general motor performance [31,32]. The Bayley Scales are frequently applied clinical measures of infant development with a good reliability and validity (assessment: about 20 min).

The three measurements partially overlap, but each of the assessments provides its own type of information: AIMS and GMFM on gross motor development (AIMS appropriate for the youngest ages, GMFM for the older infants), Bayley's PDI on general motor development and IMP on the quality of motor performance. The IMP, AIMS, GMFM and neurological examination will be integrated into one assessment which lasts about 30 min.

3. Bayley Scales of Infant Development, Mental Development Index (MDI) [31,32] to measure cognitive development (assessment about 20 min)

4. Vineland Adaptive Behavior Scales (VABS). The VABS is a scoringlist which assesses by means of structured interview of caregivers functional status in communication, daily living skills, socialization and motor skills in children with or without disabilities of less than 18 years. The VABS has a good reliability and validity in children with CP [33,34].

5. Pediatric Evaluation of Disability Inventory (PEDI) [35,36] to assess adaptation to and participation in activities of daily life. The PEDI is also an assessment based on a structured interview of caregivers. It provides information on functional abilities and caregiver assistance in the domains of mobility, self care and social functioning for children with CP aged 6 months to 7 years. The PEDI has a moderate to good reliability and validity [36,37].

VABS and PEDI will be integrated into one interview which lasts about 45-60 min.

6. Infant and Toddler Quality of Life Questionnaire (ITQOL) [38,39] to assess quality of life. This is a reliable and validated questionnaire to assess health related quality of life in young children. Time to complete questionnaire about 20 min.

#### Secondary family related outcomes

1. Video-analysis of caregiver's behaviour during two daily life activities (bathing, playing) according to Mahoney et al. (Maternal Behavior Rating Scale) [40] to document caregiver's ability to tune behaviour to the child (information on parenting capacities; [41]). Bathing lasts in general 15 to 30 minutes, playing will be video-taped for 10-15 minutes.

2. Utrechtse Coping Lijst to document coping (UCL) [42]. This inventory evaluates whether parents are able to deal in a competent way with the situation of having a child with CP. Sanderman and Ormel (1992) demonstrated that the UCL has a satisfactory reliability and validity [43]. It takes about 10 minutes to complete the UCL.

3. De Nijmeegse Ouderlijke Stress Index, short version (NOSI-K) to document stress of the caregivers [44]. The NOSI-K is a concise questionnaire based on the Parent Stress Index [45]. It is a parental questionnaire for children aged at least 2 years. Data of the PERRIN project indicated that also in children < 2 years the NOSI-K can be used as a measure of parental stress (Ketelaar, personal observation). It takes about 5 minutes to complete the NOSI-K.

4. Reaction to Diagnosis Interview (RDI) [46]. The RDI is a semistructured interview to document beliefs, memories and emotional reactions to a diagnosis, such as cerebral palsy. The interview lasts about 15 minutes, has a good reliability and promising validity [47,48]. Various studies used the RDI to investigate how parents react to the diagnosis of cerebral palsy [47,49]. In L2M 0-2 the RDI will primarily be used in an explorative way: no data are available on the process of reaction to the chain of diagnoses resulting in the final diagnosis of CP. Parents of children who participate in L2M 0-2 in general will be faced with the diagnosis of the adversities around the child's birth (e.g., preterm birth, asphyxia), with the diagnosis 'at high risk for developmental problems' and finally with the diagnosis CP. Secondary, we will explore whether parents in the COPCA-group achieve a better resolution than the parents in the control group. The RDI interview will be audio-recorded and be analyzed afterwards.

5. The Measure of Processes of Care, parental and professional forms (MPOC and MPOC-SP) [50]. The MPOC is a parental questionnaire to quantify the extent to which they experience family-centeredness in the care for their child. The MPOC-SP is an equivalent questionnaire to measure the perception of service providers of the family-centeredness of care. The MPOC and MPOC-SP have sufficient reliability and validity [51,52]. It takes about 10-15 minutes to complete the MPOC-questionnaires.

6. The Family Empowerment Scale (FES). The FES is a questionnaire providing information on systems advocacy, knowledge, competence and self-efficacy. It has sufficient reliability and validity [53]. It takes less than 10 minutes to complete the FES.

7. CBS-list to document caregiver's quality of life http://statline.cbs.nl/StatWeb. It will take each parent less than 5 minutes to complete this small questionnaire.

#### Working mechanisms

In order to get a better understanding of the working mechanisms of early intervention also the following measurements are included:

1. Assessment of postural control during reaching by means of multiple surface EMG recordings and kinematics [c.f. [54,55]]. This assessment allows a precise determination of the size of the infants' repertoire of postural adjustments, the infant's ability to select a specific adjustment for a situation and his/her capacity to adapt motor output. The assessment takes about half an hour.

2. Assessment of caregiver's behaviour during two daily life activities (see above). This video-based assessment will provide information on quality and quantity of the implementation of treatment principles into daily life [40].

3. Assessment of the actual contents of COPCA and TIP sessions by means of video recordings of therapeutic sessions and activities of daily life. Videos will be analysed with the Observer program according to Blauw-Hospers et al. 2010 [25].

4. Weekly diaries: parents fill in weekly diaries on program contents and therapists will provide structured information on goals and activities of treatment sessions. The diaries take 10-15 minutes to complete.

5. Daily Activities of Infants Scale (DAIS) [56]: parents classify in a 24-hour picture-logbook the activities of their children during one 24-hour period. The DAIS supplies especially information on play position and equipment use.

### Data-analysis

The analysis of the effect of COPCA-intervention will be performed according to techniques used in previous studies, which implies amongst others the inclusion of multivariate statistics (in order to take into account effect modifiers such as preterm versus full term birth, type of brain lesion, social class, family well being), preference patterns of postural activity, developmental trajectories and variation indices [[57,58,55], unpublished data Blauw-Hospers et al.].

## Discussion

In this paper we have presented the background and design for a randomized controlled trial comparing a new intervention, COPCA, with regular physiotherapeutic intervention for infants at very high risk for cerebral palsy. We aim to assess whether the COPCA approach is more beneficial for infants at risk for cerebral palsy and their families than current interventions and to get insight in the working mechanisms and effective components of early intervention.

As mentioned above, some studies show a positive effect of early intervention on cognitive development [59,10], but it is unknown which elements of the intervention lead to cognitive improvement. Regarding motor development, there is even less evidence for efficacy and effective elements of intervention [59,2,9]. One of the explanations could be the heterogeneity in interventions, which is associated with overlap in intervention strategies between study and control groups. The overlap hampers the comparison of interventions at RCT-level. Therefore, we expect at RCT-level only minor differences in this study to the advantage of COPCA. To cope with the heterogeneity in physiotherapy, the study includes detailed process evaluation on the basis of quantification of videorecordings of physiotherapeutic sessions. This allows for the assessment of the effectiveness of specific components of physiotherapeutic sessions in order to assess the effectiveness of specific components of the intervention. Recent studies using this approach showed positive associations between COPCA-related physiotherapeutical actions and developmental outcome at the age of 18 months, especially in infants who developed CP [16] (unpublished data Blauw-Hospers et al.).

We hypothesize that in our study in infants at very high risk for CP, physiotherapeutic actions based on the COPCA approach, are related to better outcomes, both in terms of child-related items and family related items. It is already known that interventions in preterm infants directed at parent child interaction have a positive effect on motor, cognitive and neurobehavioural outcome [60,61]. By focusing on family related items, caregiver child interaction may be improved and this may influence cognitive and motor development. The focus on family autonomy is supposed to support families in their own decision making processes and may improve educational skills and coping strategies to deal with the situation. In our study, we combine a family centered approach with a motor approach. Based on previous studies and theoretical frameworks, we expect COPCA or COPCA-based actions for the child to have a positive effect on cognitive, motor and daily functioning. The evaluation of working mechanisms, e.g. changes in postural control and presence of specific daily life activities, will provide further clues on the understanding of physiotherapy. This means that no matter what the outcome of the project on the level of the RCT will be, it will improve our understanding of early intervention in children with cerebral palsy. Such knowledge is a prerequisite for tailor-made guidance for children with CP and their families.

## Abbreviations

L2M: Learn 2 Move; CP: Cerebral Palsy; COPCA: Coping with and Caring for Infants with special needs - a family centered program; VIP: Vroegtijdig Interventie Project; NGST: Neuronal Group Selection Theory; TIP: Traditional Infant Physiotherapy; UMCG: University Medical Center Groningen; RCT: Randomized Controlled Trial; CA: corrected age; IMP: Infant Motor Profile; SD: Standard Deviation; NDT: NeuroDevelopmental Treatment; AIMS: Alberta Infant Motor Scales; GMFM: Gross Motor Function Measure; BSID: Bayley Scales of Infant Development; MDI: Mental Developmental Index; PDI: Psychomotor Developmental Index; VABS: Vineland Adaptive Behaviour Scales; PEDI: Pediatric Evaluation of Disability Inventory; ITQOL: Infant and Toddler Quality of Life Questionnaire; UCL: Utrechtse Coping List; NOSI-K: Nijmeegse Ouderlijke Stress Index - short version; RDI: Reaction to Diagnosis Interview; MPOC: Measure of Processes of Care; MPOC-SP: Measure of Processes of Care for Service Providers; FES: Family Empowerment Scale; CBS: Centraal Bureau voor de Statistiek; EMG: Electromyogram; DAIS: Daily Activities of Infants Scale

## Competing interests

The authors declare that they have no competing interests.

## Authors' contributions

MHA, CGBM, TD, HARM, AFB, LvDS, JMAV and CV set up the study design and participate in monitoring the study. TD performs randomization and coordinates contacts with physiotherapists and caregivers. TH, EGH and MHA carry out the assessments. TH, EGH, HARM and MHA drafted the manuscript. All authors read and approved the final.

## Pre-publication history

The pre-publication history for this paper can be accessed here:

http://www.biomedcentral.com/1471-2431/10/76/prepub
